# Mechanistic Evidence Mapping Ochratoxin A Toxicity onto Alzheimer’s Disease-Relevant Neurodegenerative Pathways: A Systematic Review of Experimental Models

**DOI:** 10.3390/toxics14070549

**Published:** 2026-06-24

**Authors:** Raquel Penalva-Olcina, Felipe Franco-Campos, Mercedes Taroncher, María-José Ruiz, Mónica Fernández-Franzón

**Affiliations:** 1 Toxicology and Food Chemistry Laboratory, Faculty of Pharmacy and Food Science, Universitat de València, Av. Vicent Andrés Estellés 22, 46100 Burjassot, Spain; raquel.penalva-olcina@uv.es (R.P.-O.); mercedes.taroncher@uv.es (M.T.); m.jose.ruiz@uv.es (M.-J.R.); monica.fernandez@uv.es (M.F.-F.); 2 Research Group in Alternative Methods for Determining Toxics Effects and Risk Assessment of Contaminants and Mixtures (RiskTox), Universitat de València, Av. Vicent Andrés Estellés 22, 46100 Burjassot, Spain

**Keywords:** ochratoxin A (OTA), Alzheimer’s disease (AD), neurotoxicity, neuroinflammation, blood–brain barrier (BBB), neurodegenerative diseases

## Abstract

Ochratoxin A (OTA) is a prevalent foodborne mycotoxin that has been increasingly recognized as a potential environmental contributor to neurodegenerative diseases. Despite extensive research, a systematic integration of how OTA replicates the specific pathological hallmarks of Alzheimer’s Disease (AD) is currently lacking. This study provides a comprehensive systematic review of the mechanistic evidence linking OTA exposure to AD-related pathways, utilizing the Adverse Outcome Pathway (AOP) framework to categorize complex toxicological data into biological key events (KEs). A systematic literature search was conducted across PubMed, Scopus, and Web of Science. A total of 24 peer-reviewed articles were selected for synthesis, comprising 14 in vitro studies and 10 in vivo investigations. The integrated evidence demonstrates that OTA exposure triggers a robust toxicological cascade that replicates several key mechanistic pathways associated with AD in experimental models. Early molecular triggers involve significant redox imbalance and mitochondrial bioenergetic failure, which serve as catalysts for sustained neuroinflammation and microglial activation. In vivo data, from multiple animal models, consistently show that these cellular dysfunctions culminate in structural damage. This systematic integration provides a clearer roadmap for future risk assessment and emphasizes the urgent need for refined regulatory guidelines to protect neurological health from chronic mycotoxin exposure.

## 1. Introduction

Neurodegenerative disorders represent a growing global health challenge, with Alzheimer’s disease (AD) being the most prevalent cause of dementia worldwide [1,2]. AD is a complex, multifactorial neurodegenerative disorder characterized by progressive decline in cognitive functions, resulting from the convergence of interconnected molecular and cellular dysfunctions [1].

Beyond the classical hallmarks of amyloid-β deposition and tau hyperphosphorylation, extensive evidence indicates that oxidative stress, through its damaging effects on lipids, proteins, and DNA and its ability to impair mitochondrial function, plays a central role in AD progression. These alterations lead to impaired neuronal energy metabolism and increase cellular vulnerability to degeneration [3]. Chronic neuroinflammation, driven primarily by activated microglia and astrocytes, further exacerbates neuronal damage through the sustained release of proinflammatory cytokines and reactive oxygen species [4,5].

In parallel, synaptic dysfunction and loss are pathological correlates of cognitive impairment, reflected in disrupted neurotransmission and altered neuronal connectivity that compromise circuits critical for learning and memory [6]. Blood–brain barrier (BBB) dysfunction and cerebrovascular alterations have also emerged as key contributors to AD pathophysiology, increasing barrier permeability and facilitating the entry of peripheral inflammatory mediators and potentially toxic compounds into the central nervous system (CNS) [7,8]. Ultimately, the cumulative impact of these processes drives neuronal apoptosis, impaired neurogenesis, and progressive neurodegeneration, thereby underscoring the complex mechanistic landscape of AD and highlighting multiple potential points of interaction with environmental neurotoxicants.

While age and genetic predisposition are well-established risk factors, growing attention has focused on the contribution of environmental exposures to neurodegenerative vulnerability. Among these, food-borne mycotoxins are of concern due to their widespread occurrence and chronic low-dose exposure in human populations. Ochratoxin A (OTA), a secondary metabolite produced by several *Aspergillus* and *Penicillium* species, is one of the most frequently detected mycotoxins in cereals, coffee, wine, and other staple foods [9]. Although OTA is classically recognized for its nephrotoxic and carcinogenic properties, accumulating experimental evidence suggests that OTA may also exert neurotoxic effects, acting on molecular and cellular pathways that overlap with those implicated in AD-related neurodegeneration [10,11].

In addition to mechanistic in vitro and in vivo studies, the relevance of OTA-induced neurotoxicity critically depends on its ability to cross the BBB and reach the CNS following systemic exposure. In this context, recent toxicokinetic and analytical investigations provide important complementary evidence supporting CNS exposure to OTA, strengthening the biological plausibility of its involvement in neurodegenerative processes [12,13].

In this context, advanced analytical methods (LC-MS/MS) have enabled the detection of OTA levels in brain tissue in chronic toxicological studies in BALB/c mice. Complementarily, cerebral microdialysis studies in awake, freely moving rats have demonstrated the presence of OTA in microdialysates following systemic administration, confirming its distribution within the brain extracellular space [14]. Finally, human biomonitoring data indicating widespread exposure helps contextualize these experimental findings and their potential relevance to neuronal health. In a prevalence study of 236 patients diagnosed with myalgic encephalomyelitis/chronic fatigue syndrome (ME/CFS) who reported chronic exposure to mold-contaminated environments, mycotoxins were frequently detected, with OTA was identified as the most prevalent compound. ME/CFS is characterized by persistent cognitive impairment, fatigue, sleep disturbances, and neuroimmune dysfunction, symptoms that overlap mechanistically with processes such as oxidative stress [15].

Although ME/CFS is distinct from AD and no causal link or direct extrapolation can be made to AD, such results exclusively serve to document that OTA can accumulate in human cohorts presenting with CFS, thereby warranting the clinical investigation of its potential role in other neurodegenerative conditions.

This review systematically synthesizes the experimental evidence on OTA neurotoxicity, focusing on molecular and cellular mechanisms relevant to neurodegeneration that are analogous to those described in AD. By integrating in vitro and in vivo findings within a framework based on Key Event (KE) and Adverse Outcome Pathway (AOP), this work identifies convergent biological processes modulated by OTA exposure and evaluates the mechanistic plausibility and consistency of these effects across different experimental models.

## 2. Materials and Methods

This study was conducted as a systematic review in accordance with PRISMA (Preferred Reporting Items for Systematic Reviews and Meta-Analyses) 2020 guidelines. The objective was to identify, appraise, and synthesize experimental evidence on OTA exposure in relation to signaling pathways and neurodegenerative mechanisms observed and similar to those that occur in AD. Due to the heterogeneity inherent in the study designs and outcome measures, a qualitative (narrative) synthesis was undertaken; no meta-analysis was performed.

### 2.1. Database Information

A comprehensive literature search was conducted in PubMed, Web of Science and Scopus for studies published between 2015 and 2026. Reference lists of selected articles and relevant reviews were manually screened to identify potentially eligible studies not captured through database searches. No study registries (e.g., clinical trial registries) were searched, as the review focused primarily on experimental in vitro and in vivo studies. References published before 2015 were considered only for background information and were not included in the systematic evidence synthesis.

### 2.2. Search Strategy

The search strategy combined controlled vocabulary terms (e.g., MeSH/Entrée, when applicable) and free-text keywords related to OTA exposure and neurodegeneration. The main search terms were structured as (“ochratoxin A” OR “OTA”) AND (“Alzheimer” OR “neurodegenerative” OR “cognitive impairment” OR “amyloid beta” OR “tau” OR “oxidative stress” OR “neurotoxicity”). Boolean operators (AND/OR) were used to refine the search. Search strategies were adapted to each database’ syntax and indexing.

### 2.3. Eligibility Criteria

#### 2.3.1. Inclusion Criteria

A 10-year publication window (2015–2026) was defined as an eligibility criterion to capture contemporary toxicological approaches and mechanistic evidence relevant to current environmental health and risk assessment frameworks.

Studies were included if they met the following criteria:

1. Original research articles, including in vitro and in vivo experimental designs.
2. Studies investigating OTA exposure and its association with:
  ○ AD neuropathological markers (e.g., amyloid-β, tau phosphorylation).
  ○ Molecular and cellular signaling pathways implicated in neurodegenerative processes, including pathways associated with oxidative stress, mitochondrial dysfunction, neuroinflammation, impaired proteostasis, neuronal cell death, synaptic dysfunction, or BBB impairment.
3. Articles published in peer-reviewed journals.

#### 2.3.2. Exclusion Criteria

Studies were excluded if they:

1. Were review articles, conference abstracts, commentaries, editorials, or book chapters.
2. Focused primarily on mycotoxins other than OTA and did not report OTA-specific outcomes.
3. Investigated exclusively non-neurological endpoints (e.g., renal or hepatic toxicity) without relevance to neurotoxicity or neurodegenerative processes.
4. Lacked sufficient methodological information or relevant outcome data to allow assessment of study quality, interpretation of results, or data extraction.

Review articles were excluded from the final evidence synthesis but were screened manually to identify additional eligible primary studies through their reference lists.

### 2.4. Study Selection Process

Records identified through database searching were exported and duplicates were removed prior to screening. Titles and abstracts were independently screened by the reviewers to assess relevance according to the predefined eligibility criteria. All screening was performed manually, and no automation tools were used.

**Figure 1:**
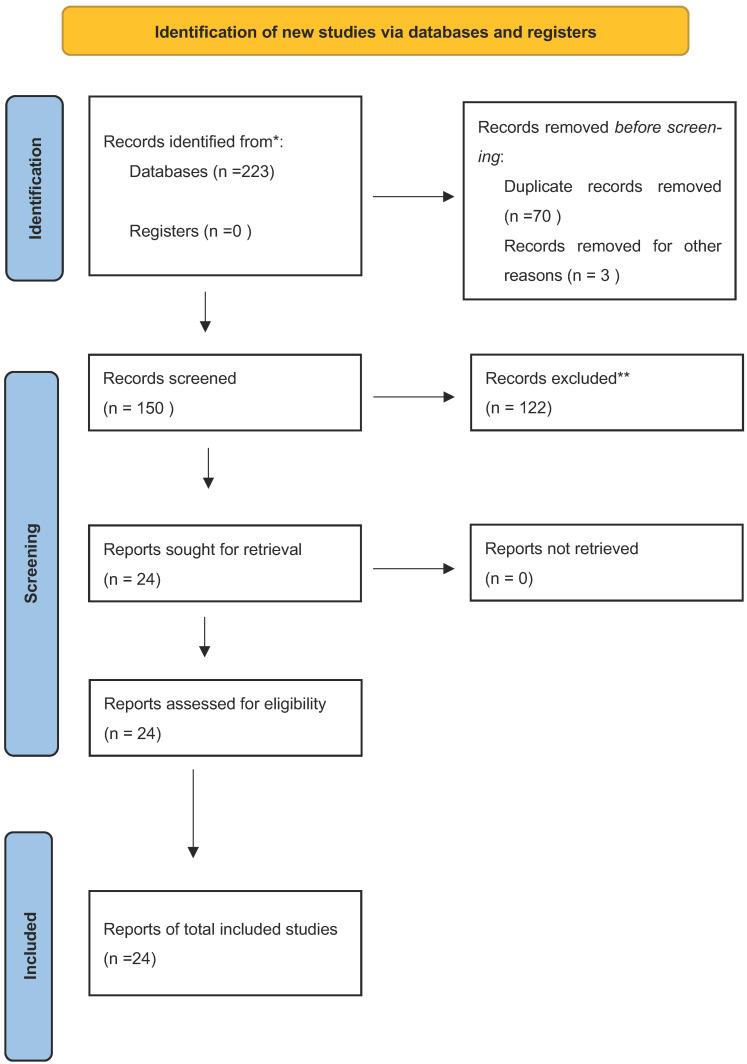
PRISMA 2020 flow diagram of study selection for the systematic review, illustrating the identification, screening, eligibility, and inclusion of studies on ochratoxin A exposure and Alzheimer’s disease-related neurodegenerative mechanisms. * Databases: Scopus (n = 98), Web of Science (n = 85), and PubMed/MEDLINE (n = 40).** Records excluded after title and abstract screening because they did not meet the inclusion criteria.

After title and abstract screening, full-text articles were retrieved and assessed for eligibility. The study selection process was documented using a PRISMA 2020 flow diagram (Figure 1).

### 2.5. Data Collection Process

Data were extracted manually from each eligible study using a standardized approach. Extracted information included:

- Experimental model (cell line, animal species/strain, or human population);
- Brain region or biological system evaluated;
- OTA dose, route, and duration of exposure;
- Key mechanistic findings and associated endpoint;
- Neurodegenerative pathways relevant to AD.

## 3. Results

### 3.1. OTA’s Induced Key Events That Reproduce Signaling Pathways Characteristic of Neurodegenerative Processes

**Figure 2:**
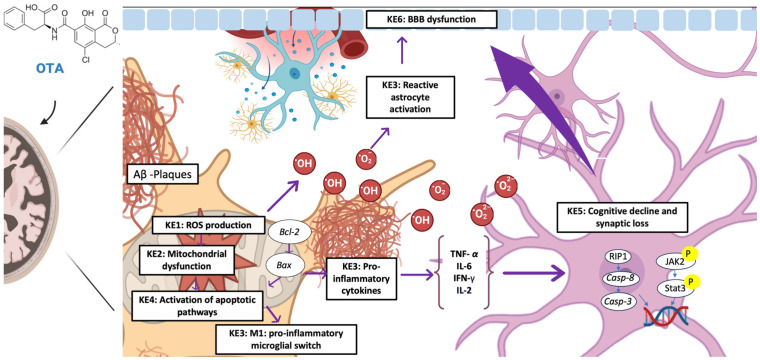
Proposed Adverse Outcome Pathway (AOP) for OTA-induced neurotoxicity and its relevance to AD-related pathways. Created in BioRender. Penalva, R. https://BioRender.com/dvsc1ii (accessed on 20 June 2026).

Based on the mechanistic evidence identified in this systematic review, OTA-induced neurotoxicity can be classified into a series of KEs that mirror critical stages of neurodegenerative diseases, including AD (Table 1). In order to facilitate a structured understanding of these processes, these findings have been integrated into a proposed Adverse Outcome Pathway (AOP) framework, as illustrated in Figure 2. This conceptual map serves as the basis for the systematic evaluation of the in vitro and in vivo evidence discussed in the following sections.

At the end of the identification and screening process, a total of 24 articles were selected from the systematic research. More specifically 14 in vitro (Table 2) and 10 in vivo studies (Table 3) were retained. In Tables, KEs were linked to shared AD-related pathways in the tables to contextualize individual mechanistic findings within broader neurodegenerative processes, enabling alignment between experimental outcomes, KE/AOP classification, and disease-relevant biological pathways.

**Figure 3:**
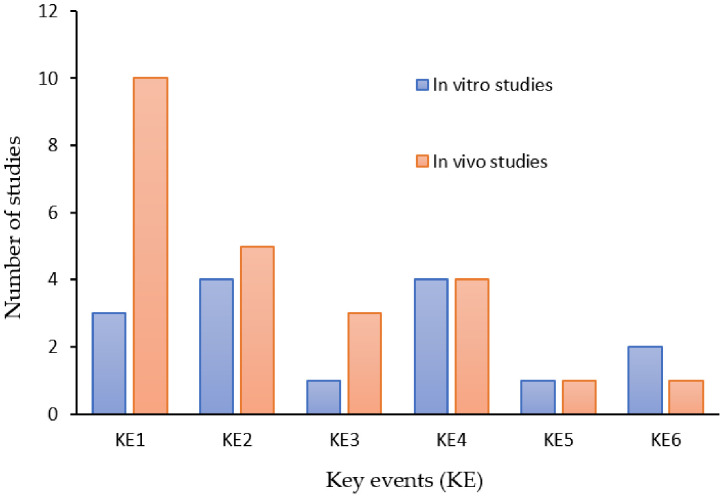
Frequency of Alzheimer’s disease-relevant key events (KEs) reported across in vitro (n = 14) and in vivo (n = 10) studies investigating OTA exposure. Bars represent the number of studies reporting each KE.

To enable a structured and transparent synthesis of the heterogeneous experimental evidence, mechanistic findings from the included studies were systematically mapped onto similar AD-relevant KEs within an AOP-oriented framework (as shown in Table 1), as performed by [38]. For each in vitro and in vivo study, reported molecular, cellular, and functional endpoints were assigned to one or more KEs according to their biological effect. Based on this classification, the frequency of each KE was quantified separately for in vitro and in vivo models and visualized as bar charts (Figure 3). These graphical representations reflect the distribution of reported KEs across the included studies and were used to identify patterns of mechanistic convergence and gaps in endpoint coverage.

It must be emphasized, however, that the frequencies illustrated in Figure 3 reflect the historical distribution of research focus and available evidence in the literature, at the period of time selected in the literature, rather than an objective measure of biological hierarchy or importance. For instance, the high prevalence of reported KE1 (oxidative stress) likely reflects the large number of experimental studies investigating redox imbalance as a central mechanism of OTA neurotoxicity. In contrast, complex structural endpoints such as BBB dysfunction (KE6) remain underrepresented, with only one in vivo study identified in this review reporting BBB-related alterations. This limited representation likely reflects the greater methodological complexity associated with evaluating BBB integrity and cerebrovascular changes in experimental models.

### 3.2. In Vitro Neurotoxic Mechanisms of OTA Similar to AD-Associated Pathways

The neurotoxic potential of OTA was evaluated through a systematic review of in vitro studies, mapping observed cellular alterations onto a sequence of interconnected KEs associated with neurodegenerative processes (Table 2). The distribution of these studies (Figure 2) indicates a predominant focus on oxidative and nitrosative stress (KE1), which represents the most extensively documented mechanism, followed by mitochondrial dysfunction (KE2) and apoptotic signaling pathways (KE4).

OTA-induced toxicity is initiated by a marked redox imbalance (KE1), consistently observed across multiple neuronal and non-neuronal models. This early disruption is closely linked to mitochondrial impairment (KE2), including alterations in bioenergetics, mitochondrial membrane potential, and calcium homeostasis. These processes converge on the activation of apoptotic pathways (KE4), characterized by caspase activation, DNA damage, and modulation of pro- and anti-apoptotic proteins, ultimately leading to programmed cell death [38].

In parallel, neuroinflammatory activation (KE3) is supported by increased production of pro-inflammatory cytokines in both microglial and neuronal models, indicating that OTA promotes a pro-inflammatory cellular environment (Table 2). Synaptic dysfunction and neurotrophic impairment (KE5), reflected by alterations in neuronal markers such as AChE, BDNF, and TH, further suggest disruption of neurotransmission and neuronal survival [19,27]. Regarding BBB dysfunction (KE6), this represents a potentially relevant late-stage event, as available in vitro models demonstrate compromised endothelial integrity and increased permeability, linking early molecular damage to potential neurovascular dysfunction. However, the currently available evidence remains limited, with only a small number of studies specifically addressing BBB-related endpoints. Therefore, the apparent low frequency of KE6 should not be interpreted as evidence of limited biological relevance. Rather, it likely reflects the scarcity of published data and the methodological challenges associated with evaluating BBB integrity. Based on the available evidence, it can be hypothesized that BBB dysfunction may constitute an important downstream consequence of OTA-induced neurotoxicity, although additional experimental studies are required to clarify its mechanistic contribution.

#### 3.2.1. KE1: Oxidative and Nitrosative Stress

Oxidative and nitrosative stress emerged as a prominent KEs across multiple in vitro models following OTA exposure (Table 2). This process was assessed using a range of experimental assays, including the detection of reactive oxygen (ROS) and reactive nitrogen species (RNS), evaluation of antioxidant defense systems, such as reduced glutathione (GSH) levels and glutathione peroxidase (GPx), superoxide dismutase (SOD) and catalase (CAT) activities, and the quantification of oxidative damage by lipid peroxidation (LPO) to lipids, proteins, and DNA. Together, these endpoints provided consistent evidence of OTA-induced redox imbalance at the cellular level.

ROS production was assessed using different assay types based on oxidation-sensitive fluorescent dyes, such as dihydroethidium (DHE) and dichlorofluorescein diacetate (DCFH-DA); lipid hydroperoxides were also quantified by TBARS method. In the studies conducted thus far, across exposure concentrations and time points, intracellular ROS increased consistently.

OTA-induced oxidative stress in SH-SY5Y human neuroblastoma cells and HT-22 mouse hippocampal neuronal cells was studied [17,24,25,29]. Cells were exposed for 24 h to OTA at 2.5–30 µM and intracellular ROS levels were assessed using the DHE fluorescent probe assay. OTA exposure resulted in a significant increase in ROS relative to controls, with ~1.5–1.6-fold elevation at 10 µM OTA after 24 h in both cell lines [17,24,25,29]. In parallel, complementary mechanistic evidence was reported in the same SH-SY5Y cell model. Specifically, exposure to OTA at 0.8–3.1 µM increased ROS production, reaching approximately 2.0-fold and 2.4-fold higher levels than the control after 15 and 45 min of exposure, respectively, as determined by the DCFH-DA assay [25].

Importantly, only one study included in this review employed a three-dimensional (3D) neuronal model (SH-SY5Y spheroids) [29]. While most available evidence derives from conventional monolayer cultures, 3D systems more closely mimic the structural and functional characteristics of neural tissue. The observed concentration-dependent cytotoxicity in this model supports the translational relevance of OTA-induced cellular stress and highlights the need for further studies using advanced experimental platforms to better characterize oxidative stress and downstream neurodegenerative mechanisms.

Consistent findings were also observed in Neuro-2a mouse neuroblastoma cells. It was demonstrated that exposure to OTA at 100–500 nM for 24 h resulted in a dose-dependent increase in intracellular ROS levels, measured with the DCFH-DA fluorescent probe relative to control [19]. These results indicate that OTA-induced ROS generation occurs across a broad concentration range.

Beyond neuronal models, oxidative stress responses were also observed in non-neuronal cells relevant to CNS homeostasis. In an in vitro BBB endothelial model using ECV304 cells, an increase in intracellular ROS following exposure to 100 nM OTA was reported. ROS production was quantified using DCFH-DA fluorescence at early post-exposure time points ranging from 2 to 4 h, indicating that OTA can elicit rapid redox responses in endothelial cells involved in BBB function [16].

OTA exposure (5–15 μM) induced a marked increase in intracellular ROS within 24 h in astrocytes. Specifically, ROS levels increased significantly in a dose-dependent manner, peaking at 15 μM [21].

Additional evidence of OTA-induced oxidative stress was observed in RGC-5 and SH-SY5Y cells, where OTA exposure significantly increased intracellular ROS levels compared with controls [22,24]. These findings further support oxidative stress as a common early event across different neural cell models.

ROS and NO signaling are tightly interconnected components of cellular redox homeostasis, and alterations in one pathway frequently influence the other. Increased ROS production can upregulate inducible nitric oxide synthase (iNOS) expression, leading to enhanced NO generation, while elevated NO levels can further propagate oxidative stress through the formation of RNS, such as peroxynitrite (ONOO^−^) [20].

In addition to increased ROS production, multiple in vitro studies have assessed the impact of OTA on cellular antioxidant defense systems by measuring the activities or levels of key antioxidant enzymes and redox-related molecules. These endpoints provide complementary information on cell capacity to counteract OTA-induced oxidative stress and are typically quantified using biochemical and enzymatic assays across diverse experimental models.

In Neuro-2a mouse neuroblastoma cells, the effects of OTA on cellular antioxidant defense systems were assessed following 24 h of exposure. OTA treatment resulted in a significant reduction in the activity of key antioxidant enzymes, including SOD and CAT, compared with control cells. In parallel, the activities of GPx and GR were also markedly decreased, indicating impairment of GSH-dependent antioxidant defenses [22]. Consistent with these enzymatic alterations, OTA exposure has also been associated with disruptions in intracellular redox homeostasis and mitochondrial-related oxidative imbalance [21].

Similarly, in GHA cells exposed to OTA at 5, 10, and 15 µM for 24 h, a marked reduction in intracellular GSH content was observed, quantified by CMF fluorescence. GSH levels decreased by 46%, 63%, and 79%, respectively, in a concentration-dependent manner [21]. These findings are in line with previous evidence demonstrating that OTA compromises antioxidant defenses across different neural cell models, reinforcing the role of redox dysregulation as a central mechanism of OTA-induced cytotoxicity.

#### 3.2.2. KE2: Mitochondrial Dysfunction

Exposure to OTA precipitates widespread mitochondrial dysfunction across diverse neural and vascular models of the CNS, acting as a key contributor to OTA-mediated oxidative damage (Table 2).

In human ECV304 endothelial cells were used to generate an in vitro simulation model of the BBB, OTA exposure at 100 nM elicits deleterious transcriptional modifications in genes governing mitochondrial bioenergetics. Specifically, the downregulation of subunits within complex IV of the electron transport chain (ETC) suggests a compromised capacity for oxidative phosphorylation. This was accompanied by the up-regulation of thioredoxin-interacting protein (TXNIP), a regulator of redox imbalance and mitochondrial stress, further supporting that OTA impairs metabolic competence and transcriptional regulation within the brain microvascular endothelium [16].

In human GHA astrocytes, OTA exposure (5–30 µM) triggers robust cellular defense mechanisms, notably the activation of the Nrf2 signaling pathway and its downstream antioxidant effectors, such as heme oxygenase-1 (HO-1) and NAD(P)H: quinone oxidoreductase 1 (NQO1). This adaptive response reflects an attempt to restore mitochondrial and redox homeostasis and promote mitochondrial biogenesis in response to mycotoxin-induced injury. Collectively, these data support a convergent cytotoxic mechanism in which OTA-induced neurotoxicity is driven by the disruption of mitochondrial bioenergetics and redox signaling across multiple cell lineages essential to neurological function [21].

In Neuro-2a cells, OTA exposure (100–500 nM, 24 h) significantly decreased mitochondrial membrane potential (Ψm) compared with controls, indicating mitochondrial dysfunction. This effect was accompanied by increased oxidative stress, suggesting a close relationship between redox imbalance and mitochondrial impairment in OTA-exposed cells [19].

In human astrocytic NHA-SV40LT cells, OTA exposure 0, 1, 2, 5 and 10 μM for 24 and 48 h exhibited significant increases in intracellular Ca^2+^ levels, with pronounced accumulation within the mitochondrial compartment. Ca^2+^ levels were assessed using calcium-sensitive fluorescent probes, demonstrating that OTA induced Ca^2+^ overload in astrocytic cells under these conditions [23]. Under these conditions, OTA produces a disruption of the ΔΨm, which was also observed and reported in a dose-dependent loss of ΔΨm in Neuro-2a cells after 24 h of exposure [19]. Specifically, at 500 nM, a significant decrease in ΔΨm was accompanied by a substantial increase in oxidative stress markers, including intracellular ROS and lipid peroxidation (MDA), which increased by ~40–50% compared to control conditions.

These findings indicate that OTA disrupts mitochondrial integrity through mechanisms tightly linked to oxidative stress, as further supported by the protective effects of antioxidant intervention. OTA-induced alterations in mitochondrial function and redox homeostasis were studied, reinforcing the central role of mitochondrial dysfunction in OTA-mediated toxicity [22].

#### 3.2.3. KE3: Neuroinflammation Activation

OTA acts as a potent driver of neuroinflammatory cascades, inducing a robust pro-inflammatory immune response in murine and human neural models (Table 2). In murine BV-2 microglial cells, it was demonstrated that OTA at 0.05–2 µM (sub- to low micromolar exposure concentrations) significant upregulation of pro-inflammatory cytokines, including IL-6, TNF-α, and IL-1β [20]. These findings demonstrate OTA’s capacity to activate microglial inflammatory signaling pathways, thereby establishing an in vitro neuroinflammatory environment that may mimic key pathological features of the CNS. Similar results were observed in immune cell-based/ex vivo models [26] and in human microglia-SV40 [28].

In parallel, observations in human SH-SY5Y neuroblastoma cells further substantiate this neuroinflammatory potential, as exposure to micromolar concentrations of OTA (3.125–12.5 µM) over 24–48 h, results in a dose-dependent increase in the production of IL-6 and TNF-α relative to controls. This elevated cytokine profile in neuronal cells is accompanied by cell-cycle alterations, suggesting that OTA-induced cellular stress and genomic instability are intrinsically linked to the activation of pro-inflammatory pathways [24]. Consequently, the convergence of evidence from both microglial and neuronal lineages supports that OTA acts not only as a direct cytotoxin but also as a driver of neuroinflammation, promoting a pro-inflammatory signaling milieu that may exacerbate neurotoxicity and compromise cellular survival [28].

#### 3.2.4. KE4: Apoptotic and Cell Death Signaling

Evidence from various neural and glial models, including SH-SY5Y, HT22, and GHA cells, demonstrates that OTA exposure induces apoptosis via activation of apoptotic signaling networks (Table 2). In SH-SY5Y and HT22 cells, exposure to 2.5 to 30 µM for 24 to 72 h were associated with the upregulation of p53 and DNA damage-linked responses [17,27]. In Neuro-2a cells, OTA exposure (100–500 nM, 24 h) increased the expression of cleaved caspase-3, indicating activation of the apoptotic machinery in parallel with oxidative stress and mitochondrial dysfunction [19]. Similarly, in HT22 model, OTA-induced apoptosis is primarily mediated, at least in part, through the JNK3/c-Jun signaling pathway. Specifically, at 500 nM, Western blot analysis showed a marked elevation in pro-apoptotic markers—including c-Jun, JNK3, and cleaved caspase-3 (casp-3)—with increases exceeding ~2-fold for some protein expression. This apoptotic cascade is closely linked to genomic instability, as Comet assays demonstrated a dose-dependent increase in DNA fragmentation and “tail moment” values as concentrations rose from 50 to 750 nM [25].

In GHA cells, OTA-induced cell death proceeds via the intrinsic mitochondrial cascade. This process is characterized by a significant shift in the Bax/Bcl-2 ratio, which increased Bax expression and a corresponding decrease in Bcl-2 that facilitate the activation of caspase-9 (casp-9) and the subsequent cleavage of the effector casp-3 [21].

#### 3.2.5. KE5: Synaptic Dysfunction and Neurotrophic Impairment

Regarding cholinergic dysfunction and other neuronal biomarkers, OTA significantly disrupts the neurochemical and transcriptional profiles of Neuro-2a cells after 24-h exposure (Table 2). The researchers used RT-qPCR (reverse transcription quantitative PCR) to analyze the expression of key neuronal genes and found that OTA induces a dose-dependent downregulation of AChE relative to control, a critical enzyme for cholinergic signaling. This reduction in AChE expression is considered an indicator of neurotoxicity and may contribute to the development of neurodegenerative conditions like AD [19].

In addition to cholinergic markers, the study reported a significant decrease in other essential neuronal biomarkers at 500 nM. The expression levels of BDNF and TH, the latter being a key enzyme for dopamine synthesis, were markedly downregulated in response to the oxidative stress and LPO triggered by OTA. These results suggest that OTA does not just cause cell death, but also specifically impairs the molecular machinery required for neurotransmission and neuronal survival [19]. Similar results were found in SH-SY5Y cells [27].

#### 3.2.6. KE6: BBB Dysfunction

Regarding the impact of OTA on the neurovascular unit (KE6), in vitro assessments using BBB models indicate significant alterations in endothelial integrity and metabolic function (Table 2). In PBCEC model, OTA exposure (1–10 µM) induced a dose-dependent increase in paracellular permeability and a reduction in barrier tightness [18].

These findings are complemented by transcriptomic data from ECV304 cells, where OTA at sub-cytotoxic concentrations (100 nM) triggered the upregulation of TXNIP and the downregulation of mitochondrial complex IV subunits. These molecular changes suggest that OTA induces a cellular barrier for permeability and transport compromise by a combination of redox imbalance and bioenergetic failure within the microvascular endothelium potentially facilitating the influx of systemic neurotoxicants [16]. However, ECV304 cells have been reclassified as a bladder carcinoma-derived cell line rather than a genuine BBB endothelial model. Therefore, although the observed metabolic and oxidative alterations remain informative, their relevance to neurovascular barrier function should be interpreted with caution.

### 3.3. In Vivo Neurotoxic Mechanisms of OTA Relevant to AD-Associated Pathways

The neurotoxic potential of OTA was evaluated through a systematic review of in vivo studies by mapping observed effects onto a sequence of interconnected KEs that culminate in cognitive and motor impairments (Table 3). The distribution of these studies (Figure 3) highlights a predominant focus on early-to-mid-stage cellular disruptions, particularly mitochondrial dysfunction (KE2) and apoptotic signaling and cell death pathways (KE4), each supported by four independent studies.

The initial phase of toxicity is characterized by oxidative stress (KE1), which is followed by synaptic dysfunction and neurotrophic impairment (KE5). The phase corresponding to KE1 is well-documented, with significant redox imbalance and programmed cell death reported across multiple brain regions and species. Although neuroinflammatory activation (KE3) and the final BBB dysfunction (KE6) are less frequently reported in the current dataset (one study each), they are critical structural and functional junctions that translate molecular damage into observable effects in animals.

#### 3.3.1. KE1: Oxidative and Nitrosative Stress

Table 3 presents studies involving animal experimentation in which the induction of oxidative stress through exposure to OTA causes neuronal alterations in both mammals and fish. The in vivo neuronal loss reported is directly linked to increased oxidative stress and DNA damage, which constitute initiating events in the cascade leading to cell loss [32]. These changes result in a depleted neurogenic niche and disrupted glial architecture, ultimately increasing susceptibility to neurodegeneration [22].

The zebrafish (*Danio rerio*) is a highly efficient in vivo model for neurotoxicology due to its high genetic similarity with humans and its sensitive antioxidant defense system. In a study, exposing *D. rerio* to OTA (0.5–2.0 µM) induced significant oxidative imbalance by impairing the brain antioxidant defenses. Specifically, the mycotoxin produced a significant alteration in the activity of key antioxidant enzymes, including GPx, GR, and GST, while simultaneously depleting levels of non-protein sulfhydryls (NPSH) [35]. The disturbance of thiol-based redox homeostasis resulted in an accumulation of ROS that directly correlated with the observed locomotor dysfunction. In accordance with these findings, it was demonstrated that OTA induced dose-dependent developmental toxicity in zebrafish embryos when a significant mortality rate was observed at 517 nM (5 mg/L), the upper limit of the study [34].

#### 3.3.2. KE2: Mitochondrial Dysfunction

Mitochondrial dysfunction has been observed in different organs of mammals in animal studies (Table 3). In in vivo models, the transition from oxidative stress to irreversible cellular damage is marked by a critical breakdown in mitochondrial and proteostatic homeostasis. In the mouse retina, chronic OTA exposure leads to direct mitochondrial dysfunction, characterized by increased ROS production and a significant reduction in the activity of antioxidant enzymes such as SOD and GST, indicating impaired mitochondrial redox homeostasis [22]. In the striatal system of adult BALB/c mice, OTA-induced impairment of autophagic pathways, evidenced by reduced LAMP-2A expression, may further compromise mitochondrial quality control mechanisms, thereby favoring the persistence of dysfunctional mitochondria and neuronal injury [32].

This organelle-specific damage is closely linked to a failure in cellular “quality control” or proteostasis. In adult BALB/c mice treated with oral doses of 0.21–0.5 mg/kg for four weeks, mitochondrial distress is accompanied by a significant disruption of the autophagy-lysosome pathway. Western blot analysis in the striatal system revealed a marked decrease in LAMP-2A expression, a key protein for chaperone-mediated autophagy. This impairment was associated with the pathological accumulation of phosphorylated α-synuclein, with levels showing a significant increase compared to control groups [31].

Furthermore, the mitochondrial collapse is confirmed by the shift in the Bax/Bcl-2 ratio, which serves as the molecular switch for the intrinsic apoptotic pathway. In hippocampal and glial models, OTA induces a decrease in anti-apoptotic Bcl-2 and a corresponding increase in pro-apoptotic Bax, reaching levels that often exceed a two-fold change relative to controls in protein amount [32,39]. This structural failure of the mitochondria allows the release of pro-apoptotic factors into the cytosol, effectively preparing the cell for the enzymatic execution phase (KE4). Collectively, these findings illustrate that at doses as low as 0.21 mg/kg, OTA systematically compromises mitochondrial defenses and protein clearance mechanisms, creating a neurodegenerative environment before macroscopic anatomical lesions become visible [31].

#### 3.3.3. KE3: Neuroinflammatory Activation

In the in vivo context, OTA triggers a robust neuroinflammatory response characterized by the pathological activation of the brain’s innate immune system and a simultaneous loss of homeostatic glial support. Chronic exposure drives microglia towards a reactive phenotype, a state associated with a dramatic increase in pro-inflammatory markers. Specifically, studies have shown that OTA exposure leads to a significant increase in the secretion of cytokines such as TNF-α and IL-1β [22]. This microglial polarization is complemented by reactive astrogliosis, particularly in the hippocampus. This effect has been observed in rats administered 289 µg/kg i.p., where elevated GFAP levels, indicative of an increased astrocyte activity, were observed alongside NF-kB pathway activation, which propagates a cytotoxic neuroinflammatory microenvironment (Table 3) [30].

#### 3.3.4. KE4: Apoptotic and Cell Death Signaling

The progression from oxidative stress to tissue-level degeneration is driven by the activation of programmed cell death pathways across various in vivo models (Table 3). In the mouse hippocampus, chronic exposure to 3.5 mg/kg OTA results in a significant reduction in cell viability in a dose-dependent manner within the dentate gyrus [32]. This cellular loss is mechanistically linked to the activation of the intrinsic (mitochondrial) apoptotic pathway, in which the toxin induces an increase in the Bax/Bcl-2 ratio. The upregulation of the pro-apoptotic protein Bax, coupled with the downregulation of anti-apoptotic Bcl-2, triggers the permeabilization of the mitochondrial membrane. Consequently, the enhancement of apoptosis in this region, which is characterized by a tightly regulated balance between neuronal generation and elimination, may reflect an increased susceptibility to neurodegenerative processes in the exposed animals.

On the other hand, following the breakdown of mitochondrial integrity (KE2), there is a significant recruitment and activation of Casp-9, which subsequently cleaves and activates Casp-3. This pathway is not limited to neurons; in the mouse striatum, OTA exposure at doses of 0.21–0.5 mg/kg leads to dopaminergic neuronal dysfunction and loss, which is exacerbated by impaired autophagy and the accumulation of phosphorylated α-synuclein. The failure to clear damaged proteins through the lysosomal autophagic pathway, marked by reduced LAMP-2A levels, serves as a secondary trigger for apoptotic signaling [31].

In rat models, the activation of these cell death markers is associated with structural lesions in the hippocampus and pathological gliosis, confirming that KE4 represents the critical molecular phase where biochemical stress is converted into permanent structural damage (Table 3) [30].

#### 3.3.5. KE5: Synaptic Dysfunction and Neurotrophic Impairment

OTA exposure disrupts neuronal connectivity and survival signaling (Table 3). In mouse models, chronic exposure to 3.5 mg/kg of OTA leads to a significant reduction in cell viability within the hippocampal neurogenic niche, characterized by a depleted neurogenic niche and a dysfunctional glial architecture [32]. Similar results were obtained by Brahim et al. in the hippocampus of rats exposed to OTA at lower doses. In this study, the administration of 289 µg/kg i.p. induced hippocampal lesions, gliosis, and memory deficits linked to cholinergic dysfunction [30]. Specifically, in Zebrafish embryos, concentrations as low as 12 to 1000 nM triggered a massive oxidative stress.

#### 3.3.6. KE6: BBB Dysfunction

Exposure of embryonic zebrafish to OTA during early development induced significant cerebrovascular toxicity in a dose-dependent manner [36]. Specifically, exposure to 2.5 µM OTA resulted in a 45% increase in intracerebral hemorrhage, whereas exposure to a higher dose of 5 µM was associated with an 82% increase in disrupted cerebrovascular patterning. The study revealed that OTA dysregulates the miR-731/PRLRa axis, whereby the significant downregulation of miR-73 compromises the structural integrity of the BBB. These vascular alterations lead to increased vascular permeability, as evidenced by the infiltration of neurotoxic agents into the CNS. Ultimately, the impairment of vessel formation and the loss of BBB integrity at these concentrations significantly increase neurodevelopmental vulnerability during the first 72 h post-fertilization (hpf).

## 4. Discussion

To assess the mechanisms of OTA neurotoxicity and its potential link to neurodegenerative diseases, specifically AD, a systematic review was performed. Of the 223 articles initially retrieved, 150 were excluded after screening. The primary exclusion criteria removed studies not focused on OTA or addressing non-neurotoxic effects; review articles were also excluded to prioritize primary data. A five-year publication window was applied to ensure the inclusion of state-of-the-art methodologies, such as high-resolution proteostasis and autophagy assays, that were largely unavailable in older studies. This focus prioritizes contemporary toxicological paradigms, aligning the evidence with current environmental exposure levels and the most recent regulatory frameworks for AOP. Moreover, the search terms were designed to be inclusive, combining “OTA AND” [term1 OR term2] without applying exclusionary “NOT” operators. Finally, keywords were restricted to neurotoxicity-related terms and neural models, thereby excluding systemic OTA effects, such as nephrotoxicity or hepatotoxicity outcomes unique to brain architecture and cognitive function.

Consistent in vitro evidence demonstrates that OTA acts as a potent inducer of oxidative stress (KE1) across various neuronal models. Studies involving cell lines such as SH-SY5Y, HT22, and GHA have reported significant surges in the production of ROS, hydroxyl radicals, and NO, often accompanied by the collapse of mitochondrial membrane potential (KE2) and the depletion of essential antioxidant defenses like GSH [17,40]. This cellular oxidative landscape is corroborated by in vivo findings, where OTA perturbs antioxidant defenses in brain and sensory tissues. In zebrafish models, acute exposure results in a marked perturbation of GPx, GR, and GST, together with depletion of non-protein thiols and increased ROS, supporting that OTA-driven ROS generation is not merely a cell-culture artifact [35]. This enzymatic preturbation is further mirrored in the mouse retina, where a significant reduction in SOD activity is associated with LPO, mitochondrial fragmentation and loss of Ψm [22].

Such “oxidative exhaustion” of the brain’s microenvironment is best framed as a major early contributor in neuronal metabolic pathways similar to the ones involved in AD. In the absence of adequate enzymatic protection, the brain’s high lipid content is highly susceptible to chronic oxidation, destabilizing neuronal membranes and creating a pro-aggregant environment [39]. Furthermore, this oxidative state is closely linked to the formation of beta-amyloid (Aβ) plaques. Extensive evidence indicates that oxidative stress is an early upstream event that promotes the amyloidogenic processing of the amyloid precursor protein (APP) [41]. By inducing mitochondrial dysfunction (KE2) and sustained ROS production in neural tissues, OTA may contribute to the formation of a microenvironment that accelerates peptide misfolding. However, it must be emphasized that direct experimental evidence establishing a definitive causal link between OTA exposure and classical amyloid plaque deposition remains highly limited in the current literature. Consequently, these pathways should be interpreted cautiously as overlapping biochemical mechanisms rather than a proven direct driving force of amyloid pathology.

The resulting proteostatic disruption is evidenced by the accumulation of misfolded proteins and downregulation of the chaperone-mediated autophagy receptor LAMP-2A in the striatum, mirroring the failure of protein clearance observed in a neurodegenerative disease such as Parkinson’s [31]. Consequently, by weakening antioxidant defenses and promoting neuroinflammation, OTA exposure replicates the biochemical conditions required for the transition from soluble proteins to insoluble amyloid aggregates (senile plaques), ultimately potentially contributing to the loss of hippocampal plasticity and the cognitive decline characteristic of AD [42].

Regarding neuroinflammatory activation, OTA exposure acts as a potent amplifier of the neurodegenerative processes, mimicking the chronic inflammation that drives multiple pathological features of AD. In vitro models demonstrate that OTA induces a robust M1-like microglial activation, marked by the upregulation of isolectin B4 (IB4)-binding microglia and the sustained release of pro-inflammatory cytokines such as TNF-α and IL-1β [20,28]. IB4 is a widely used lectin marker that labels activated/ameboid microglia in brain tissue. Beyond stimulating amyloidogenic pathways, these cytokines are known to activate intracellular kinases (such as GSK-3β) that lead to the hyperphosphorylation of Tau protein [43,44].

Furthermore, the pathological astroglyosis and astrocytic NF-*κ*B activation observed in hippocampal models and rat studies [30] parallel the reactive gliosis that contributes to BBB dysfunction (KE6) in AD patients. The observed dysregulation of MT-I/II and the morphological atrophy of astrocyte processes, reported in models utilizing high experimental OTA concentrations [32], highlight a pathway that could theoretically undermine the metabolic “shield” of the neuron, increasing cellular vulnerability to peripheral toxins and impaired nutrient transport. In the pathophysiology of the AD brain, this type of glial failure is a major recognized contributor to aberrant synaptic pruning, where overactive microglia and dysfunctional astrocytes clear healthy synapses [8,45]. However, it is essential to note that while these experimental alterations provide plausible mechanistic links, they were identified under micromolar conditions that significantly exceed real-world human dietary exposures. Therefore, whether repeated exposure to nanomolar blood levels of OTA can trigger equivalent morphological astrocytic atrophy in human clinical settings remains an open question that requires lower-dose toxicological validation.

By inducing a chronic inflammatory state, OTA replicates the multifaceted pathological environment required for neurovascular unit breakdown and subsequent neuronal death (KE6). Both in vitro and in vivo evidence confirm that OTA possesses the capacity to disrupt the BBB, a critical interface that is increasingly recognized as a ‘first responder’ in AD. In cellular models such as ECV304, which has been historically utilized as a BBB proxy, OTA exposure triggers deleterious transcriptional modifications in genes governing mitochondrial bioenergetics and redox balance [16]. While these findings provide valuable evidence of OTA-induced metabolic stress within the microvascular endothelium, it is essential to acknowledge that ECV304 has been genetically re-identified as a T24 urinary bladder carcinoma derivative [45]. Therefore, while the observed mitochondrial impairment (KE2) and TXNIP-mediated stress remain mechanistically relevant, it is imperative that these results must be excluded from the database pertaining to KE6 on BBB dysfunction. Future research using primary human brain microvascular cells or iPSC-derived models is necessary to confirm how these metabolic alterations translate into physical barrier permeability and paracellular transport disruption [16,18].

To overcome the limitations of conventional monocultures, the implementation of New Approach Methodologies (NAMs) is becoming essential in neurotoxicity assessment. Recent advancements, such as the dynamic microfluidic model developed demonstrate that SH-SY5Y spheroids under physiological-like flow conditions exhibit differential sensitivity to OTA compared to static cultures [29]. This underscores the importance of incorporating shear stress and three-dimensional architecture to accurately replicate the neuron microenvironment. These cutting-edge methodologies not only enhance the translational value of in vitro data for neuronal research but also align with the global transition toward more predictive, non-animal testing strategies.

In vivo evidence from experiments on embryonic zebrafish demonstrates that OTA-induced vascular damage, including intracerebral hemorrhage and altered vascular signaling, via the miR-731/prolactin receptor axis. This highlights the mycotoxin’s potent vasculotoxicity [36]. In the context of neurodegenerative diseases similar to AD, compromised BBB function facilitates a cycle, allowing the influx of peripheral neurotoxic mediators into the brain parenchyma while also impairing the efflux of Aβ and metabolic waste [46]. In experimental systems, OTA does not merely attack neurons directly, but potentially accelerates the transition from localized oxidative stress to broader neurovascular dysfunction.

The persistence of these markers suggests that OTA exposure transitions the brain into a self-sustaining cycle of neuroinflammation. This cycle not only promotes protein aggregation but also contributes to the structural disruption of hippocampal circuits, providing a comprehensive mechanistic link between toxic exposure and the diverse clinical symptoms that define AD-related neurodegeneration, ranging from memory loss to motor dysfunction [47].

The disruption of synaptic integrity (KE5) observed in OTA models is most directly linked to the clinical symptoms of neurodegenerative diseases analogous to Alzheimer’s, where “synaptic failure” is recognized as a better predictor of cognitive decline than plaque density alone [48]. In vivo studies demonstrate that OTA exposure significantly downregulates MAP2, a microtubule-associated protein essential for dendritic structural integrity and cytoskeletal stability, which are critical for maintaining functional neuronal connectivity [30,32]. This loss of synaptic markers in the hippocampus mirrors the preclinical AD cerebrospinal fluids, where the physical “pruning” of synaptic connections between neurons leads to impairment of long-term potentiation (LTP), the cellular basis for learning and memory [49,50].

Furthermore, the neurochemical imbalance observed in OTA-treated models, specifically the reduction in ACh levels and the inhibition of AChE activity [30], reflects the “cholinergic hypothesis” of neurodegenerative processes similar to those observed in AD [51]. This reduction in cholinergic tone directly impairs hippocampal circuits responsible for memory encoding. Combined with the OTA-induced glutamate-mediated excitotoxicity and disrupted calcium signaling, this results in a state of “synaptic noise” degrading signal fidelity and impairing synaptic transmission.

Ultimately, the observed reduction in dendritic spine density and the atrophy of neuronal processes establish a structural correlation that connects molecular damage to functional impairment [32]. In the context of AD, this synaptic collapse acts as a critical juncture, mediating proteostatic disruption and neuroinflammation into the memory loss and motor incoordination observed in KE5. By dismantling the specialized junctions of neuronal communication, OTA effectively may mimic the “disconnection syndrome” that defines the progression from mild cognitive impairment to the profound dementia of the late stage that occurs in neurodegenerative diseases such as AD.

Despite the compelling mechanistic overlaps identified in this review, several critical limitations inherent to the current state of the literature must be acknowledged. First and foremost, a definitive causal link between OTA exposure and the clinical onset or progression of human AD remains to be established. The vast majority of the synthesized data originates from traditional 2D/3D in vitro platforms and acute or subchronic animal models. While these experimental setups are invaluable for hazard identification and mapping molecular cascades, they cannot fully replicate the multifactorial, long-term, and slowly progressive nature of human neurodegeneration. Concurrently, a major thematic imbalance exists within the current body of evidence, which focuses heavily on broad upstream cellular endpoints, namely oxidative stress and mitochondrial failure, while direct experimental evaluations of specific hallmark AD features, such as amyloid-β aggregation and tau hyperphosphorylation, remain critically limited. This literature gap highlights that current mycotoxin research has largely characterized generic cytotoxicity rather than disease-specific neuropathology.

Furthermore, there is a severe lack of direct human clinical evidence, as no studies to date have systematically co-localized OTA accumulation within postmortem human AD brains, nor are there long-term prospective epidemiological cohorts correlating dietary mycotoxin intake with AD diagnosis. Although human biomonitoring data in other cohorts presenting with neurocognitive symptoms, such as ME/CFS, confirm that OTA can distribute to human tissues under chronic exposure conditions, these observational findings serve exclusively as a proof-of-principle and cannot be directly extrapolated to AD pathology or diagnosis.

A critical aspect when interpreting these findings is the translational relevance of experimental exposure levels. Human biomonitoring studies report OTA plasma concentrations typically in the low nanomolar range (0.1–10 nM), reflecting chronic dietary exposure [52]. In contrast, many in vitro and in vivo studies employ micromolar concentrations, which are 100- to 1000-fold higher and may exceed typical human exposure levels. While OTA exhibits a prolonged biological half-life and strong binding to serum albumin, supporting its theoretical potential for bioaccumulation under repeated low-dose exposure conditions [53], there is currently an absence of quantitative toxicokinetic data in humans demonstrating that chronic low-dose exposure can achieve micromolar concentrations within the brain parenchyma. Consequently, while direct quantitative extrapolation remains challenging, the mechanistic pathways identified in experimental systems must be interpreted as a map of biological hazard identification rather than a direct reflection of real-world human clinical risk. This highlights the urgent need for future studies employing human-relevant, low-dose exposure paradigms and advanced experimental models capable of recapitulating chronic low-dose conditions to better understand the potential contribution of OTA to long-term neurodegenerative processes.

## 5. Conclusions

This systematic review provides a comprehensive synthesis of the neurotoxic pathways of OTA and their mechanistic convergence with the pathological hallmarks of AD. By applying an AOP framework, the evidence indicates that OTA-induced oxidative stress (KE1) and mitochondrial dysfunction (KE2) function as initiating events of a cascade leading to chronic neuroinflammation (KE3). In this context, these early upstream events should be understood as general neurotoxic alterations that establish the pathological microenvironment required to drive downstream AD-specific pathways. The evidence consistently shows that OTA replicates key biochemical conditions required for the misfolding of amyloidogenic proteins and the subsequent impairment of hippocampal neurogenesis. While direct co-localization of OTA with amyloid plaques in human subjects remains to be systematically investigated, the molecular “fingerprint” of the mycotoxin suggests it may represent a plausible environmental contributor. The ability of OTA to disrupt the BBB and impair synaptic plasticity provides a plausible mechanistic explanation in rodent and cell models. While it remains speculative whether this translates directly to human clinical settings, these findings highlight how dietary or environmental exposure could potentially exacerbate cognitive decline, emphasizing the need for human postmortem and epidemiological validation.

## Figures and Tables

**Table 1:** Classification of the key neurodegenerative events relevant to Alzheimer’s disease-related pathways.

Event	Key Processes	Relevance to AD-Related Pathways
KE1: Oxidative and nitrosative stress	- Increased intracellular ROS and RNS production - Depletion of antioxidant defenses (↓ GSH, ↓ Nrf2/HO-1 signaling) - Lipid, protein, and DNA oxidative damage	Oxidative stress are events implicated in AD, promoting macromolecular damage, disruption of cellular homeostasis, increased neuronal vulnerability, synaptic dysfunction, and progressive neurodegeneration.
KE2: Mitochondrial dysfunction	- Loss of mitochondrial membrane potential (ΔΨm) - Impaired ATP production and bioenergetic failure Dysregulated mitochondrial Ca^2+^ handling	Mitochondrial dysfunction is strongly implicated in AD and often precedes amyloid-β accumulation and tau pathology, contributing to neuronal energy failure and apoptosis.
KE3: Neuroinflammatory activation	- Microglial activation - Sustained release of pro-inflammatory mediators (IL-1β, IL-6, TNF-α, IL-18, CXCL8) - Induction of iNOS and nitric oxide production - Reactive astrocyte activation	Chronic neuroinflammation accelerates synaptic loss and neuronal damage in AD and is increasingly recognized as a driver of disease progression.
KE4: Apoptotic and cell death signaling	- Increased Bax/Bcl-2 ratio - Activation of caspase-3 and caspase-9 - p53-mediated pro-apoptotic signaling	Activation of intrinsic apoptotic pathways contributes to progressive neuronal loss observed in AD.
KE5: Synaptic dysfunction and neurotrophic impairment	- Reduced BDNF expression - MAPK and stress-activated kinase signaling - Impaired neuronal connectivity and plasticity	Synaptic dysfunction and loss of neurotrophic support are early events in AD and strongly correlate with cognitive decline.
KE6: Blood–brain barrier (BBB) dysfunction	- Endothelial cytotoxicity - Increased BBB permeability - Compromised barrier integrity	BBB disruption contributes to increased brain vulnerability, enhanced neuroinflammation, and impaired clearance of neurotoxic molecules in AD.

Abbreviations: ↑ increase; ↓ decrease.

**Table 2:** In vitro evidence of OTA-induced neurotoxic mechanisms similar to Alzheimer’s disease related pathways.

Model(s)	OTA Dose/ Exposure	Key Mechanistic Findings (In Vitro)	AOP-Aligned KEs	Shared AD-Related Pathways/AOP Context	Reference
ECV304/C6 (BBB proxy)	100 nM	↑ ROS and altered expression of mitochondrial stress	KE1: Oxidative stress; KE2: Mitochondrial dysfunction; KE6: BBB susceptibility	BBB–mitochondrial dysfunction AOPs could be relevant neurodegeneration (Note: ECV304 is a T24 derivative)	[16]
HT-22	0.5–10 µM	↑ ROS and p53	KE1: Oxidative stress; KE4: Apoptosis	Hippocampal neuronal vulnerability	[17]
PBCEC	1–10 µM	Cytotoxicity; barrier-weakening effects	KE6: BBB integrity disruption	BBB dysfunction increasing brain susceptibility to neurotoxicants	[18]
Neuro-2a	100–500 nM	↑ ROS, MDA, JNK3 and cleaved casp-3 ↓ antioxidant defenses, LPO, ΔΨm, AChE, BDNF, TH and NOS2	KE1: Oxidative stress; KE2: Mitochondrial dysfunction; KE4: Apoptosis; KE5: Cholinergic dysfunction	Cholinergic deficit and mitochondrial impairment	[19]
BV2 microglia	0.05–2 µM	↑ iNOS, NO, TNF-α, IL-1β and IL-6	KE1: Oxidative stress/Nitrosative stress; KE3: Microglial activation	Neuroinflammatory and nitrosative stress	[20]
GHA	5–30 µM	Dose-dependent cytotoxicity; ↑ ROS; ↓ GSH; activation of Bax, casp-9/-3; modulation of Nrf2/HO-1/NQO1; partial protection by NAC	KE1: Oxidative stress; KE2: Mitochondrial dysfunction; KE4: Apoptosis	Glial oxidative stress and apoptosis could contribute to neurodegenerative cascades	[21]
RGC-5	100 and 200 µg/L (3 days)	↑ ROS, MDA and apoptosis; ↓ GSH, SOD and ΔΨm	KE1: Oxidative stress; KE2: Mitochondrial dysfunction	Sustained oxidative damage promotes Aβ and tau pathology (Note: RGC-5 is a 661W derivative)	[22]
NHA-SV40LT	0, 1, 2, 5 and 10 μM for 24 and 48 h	↑ intracellular Ca^2+^; MAPK activation	KE1: Stress signaling activation KE2: Ca^2+^ homeostasis disruption	Astrocytic dysfunction contributing to neuronal vulnerability in neurodegeneration	[23]
SH-SY5Y	3.125–12.5 µM	↑ ROS, IL-6 and TNF-α	KE1: Oxidative stress; KE3: Neuroinflammatory signaling	Neuroinflammation-mediated synaptic dysfunction	[24]
SH-SY5Y	0.8, 1.5, 3.1 µM	↑ ROS ↑ Bax/Bcl-2 ratio, Casp-3 activation and MN formation	KE1: Oxidative stress KE4: Apoptotic and cell death signaling	Neuronal death could contribute to neurodegeneration	[25]
Immune cell-based/ex vivo models	n.s.	↑ TNF-α and IL-6 production	KE3: Peripheral immune activation	Peripheral–central immune crosstalk could contribute to neuroinflammation	[26]
SH-SY5Y	1–5 µM	Activation of MAPK and p53 pathways; ↑ Bax; ↓ BDNF	KE4: Stress-activated apoptotic signaling; KE5: Synaptic dysfunction	Synaptic failure and neurotrophic loss preceding cognitive decline in neurodegeneration	[27]
Human microglia-SV40	0.1–1 µM	↑ IL-1β, IL-18, CXCL8	KE3: Microglial activation; chronic neuroinflammation	Sustained microglial inflammation	[28]
SH-SY5Y 3D spheroids	6.25–100 µM (24 h)	Concentration-dependent cytotoxicity; increased dead cell fraction; variable sensitivity vs. static	KE1: Cellular stress; KE2: Mitochondrial dysfunction	Cellular loss and stress responses could be relevant to early neuronal damage	[29]

Abbreviations: AChE, acetylcholinesterase; AD, Alzheimer’s disease; AOP, adverse outcome pathway; Bax, Bcl-2-associated X protein; BBB, blood–brain barrier; Bcl-2, B-cell lymphoma 2; BDNF, brain-derived neurotrophic factor; BV2, murine microglial cell line; Ca^2+^, intracellular calcium; Casp, caspase; CXCL8, C-X-C motif chemokine ligand 8 (interleukin-8); Ψm, mitochondrial membrane potential; ECV304, human urinary bladder carcinoma cell line; GHA, human astrocyte cell line; GSH, reduced glutathione; HO-1, heme oxygenase-1; HT-22, mouse hippocampal neuronal cell line; IL, interleukin; iNOS, inducible nitric oxide synthase; JNK3, c-Jun N-terminal kinase 3; KE, key event; LPO, lipid peroxidation; MAPK, mitogen-activated protein kinase; MDA, malondialdehyde; MN: micronucleus; NAC, N-acetylcysteine; Neuro-2a, mouse neuroblastoma cell line; NHA-SV40LT, normal human astrocytes immortalized with SV40 large T antigen; NO, nitric oxide; n.s., not significant; NOS2, nitric oxide synthase 2; NQO1, NAD(P)H quinone dehydrogenase 1; Nrf2, nuclear factor erythroid 2-related factor 2; OTA, ochratoxin A; p53, tumor protein p53; PBCEC, porcine brain capillary endothelial cells; RGC, retinal ganglion cells; ROS, reactive oxygen species; SH-SY5Y, human neuroblastoma cell line; SV40, simian virus 40; TH, tyrosine hydroxylase; TNF-α, tumor necrosis factor alpha; ↑, increase; ↓, decrease.

**Table 3:** In vivo evidence of OTA-induced neurotoxic mechanisms relevant to Alzheimer’s disease related pathways.

Animal	Region	Dose of OTA	Key Mechanistic Findings (In Vivo)	AOP-Aligned KEs	Shared AD-Related Pathways	References
Mouse	Retina	100 and 200 µg/kg during 4 weeks	Mitochondrial dysfunction; increased ROS; reduced SOD and GST activity	KE1: Oxidative stress; KE2: Mitochondrial dysfunction; KE4: Apoptotic and cell death signaling	Redox imbalance contributing to neuronal vulnerability	[22]
Adult mice	Brain	Subchronic exposure (low doses)	Motor deficits; decreased dopamine levels; altered neurotransmitter signaling	KE5: Neurotransmitter dysfunction	Dopaminergic signaling impairment overlapping with neurodegenerative mechanisms	[19]
Adult Wistar rats	Hippocampus	289 µg/kg i.p., every 48 h (12 doses)	Cognitive impairment; hippocampal lesions; gliosis; reduced brain weight	KE4: Neuronal damage KE5: Cognitive impairment	Learning and memory deficits linked to hippocampal and cholinergic dysfunction	[30]
Adult BALB/c mice	Striatal system	0.21–0.5 mg/kg orally for 4 weeks	Loss of dopaminergic innervation; ↑ phosphorylated α-synuclein; ↓ LAMP-2A; impaired autophagy	KE2: Proteostasis disruption; KE4: Neuronal damage KE5: Neuronal dysfunction	Impaired autophagy and protein aggregation relevant to neurodegeneration	[31]
Mouse	Hippocampus	3.5 mg OTA/kg b.w. (i.p. single-repeated)	Increased ROS and DNA damage; reduced astrocyte density and ↓ MAP2	KE1: Oxidative stress; KE2: Mitochondrial dysfunction; KE4: Apoptotic and cell death signaling; KE5: Glial cell loss; Impaired neurogenesis	Reduced neuronal support and plasticity associated with neurodegenerative vulnerability	[32]
Mouse	SVZ	3.5 mg OTA/kg b.w. (i.p. single-repeated)	Reduced proliferation and differentiation of neural progenitor cells; decreased BrdU^+^ cells and neuroblasts	KE5: Impaired adult neurogenesis; Reduced neuronal/glial renewal	Loss of neurogenic capacity and reduced brain plasticity	[33]
Zebrafish embryos (*Danio rerio*)	Whole organism	0.16- 5 mg/L OTA in water, up to 96 hpf	Dose-dependent mortality; embryonic damage; reduced heart rate	KE1: Oxidative stress KE5: Neuronal dysfunction	Oxidative stress-driven developmental and physiological disruption	[34]
Zebrafish	Brain	Not specified	Altered antioxidant enzymes (GPx, GST, GR, NPSH) indicating redox imbalance	KE1: Oxidative stress; KE5: Neuronal dysfunction	Oxidative imbalance associated with locomotor dysfunction	[35]
Embryonic zebrafish (*Danio rerio*)	Brain/vasculature	OTA 0.5 µM From 6 hpf; effects at 72 hpf	Intracerebral hemorrhage;	KE6: BBB susceptibility	Cerebrovascular and BBB alterations contributing to CNS vulnerability	[36]
Rat	Hippocampus-related	289 µg/kg/b.w. i.p. 48 h × 12 doses	Anxiety- and depression-like behavior; Loss of brain mass and memory deficits	KE3: Neuroinflammatory activation; KE4: Apoptotic and cell death signaling; KE5: Neuronal dysfunction and cognitive impairment	Hippocampal dysfunction, oxidative stress, apoptosis, ↓ NMDA signaling	[37]

Abbreviations: BBB, blood–brain barrier; BrdU, 5-bromo-2′-deoxyuridine; b.w.; body weight; CNS, central nervous system; GPx, glutathione peroxidase; GST, glutathione S-transferase; GR, glutathione reductase; hpf, hours post-fertilization; i.p., intraperitoneal; KE, key event; LAMP-2A, lysosome-associated membrane protein 2A; MAP2, Microtubulose associated protein 2; NMDA, N-methyl-D-aspartate receptor; NPSH, non-protein sulfhydryl groups; OTA, ochratoxin A; ROS, reactive oxygen species; SOD, superoxide dismutase; SVZ, subventricular zone; ↑, increase; ↓, decrease.

## Data Availability

No new data were created or analyzed in this study. Data sharing is not applicable to this article.

## References

[1] Barrantes F.J. (2024). Cognitive synaptopathy: Synaptic and dendritic spine dysfunction in age-related cognitive disorders. Front. Aging Neurosci..

[2] Sahoo R.K., Gupta T., Smilly, Kumar V., Rani S., Gupta U., Yadav A.K., Shukla R., Flora S.J.S. (2022). Aetiology and pathophysiology of neurodegenerative disorders. Nanomedical Drug Delivery for Neurodegenerative Diseases.

[3] Yeung A.W.K., Tzvetkov N.T., Georgieva M.G., Ognyanov I.V., Kordos K., Jóźwik A., Kühl T., Perry G., Petralia M.C., Mazzon E. (2021). Reactive Oxygen Species and Their Impact in Neurodegenerative Diseases: Literature Landscape Analysis. Antioxid. Redox Signal..

[4] Shi Z.M., Han Y.W., Han X.H., Zhang K., Chang Y.N., Hu Z.M., Qi H.X., Ting C., Zhen Z., Hong W. (2016). Upstream regulators and downstream effectors of NF-κB in Alzheimer’s disease. J. Neurol. Sci..

[5] Skovira J.W., Wu J., Matyas J.J., Kumar A., Hanscom M., Kabadi S.V., Fang R., Faden A.I. (2016). Cell cycle inhibition reduces inflammatory responses, neuronal loss, and cognitive deficits induced by hypobaria exposure following traumatic brain injury. J. Neuroinflammation.

[6] Gylys K.H.E.A. (2004). Synaptic Changes in Alzheimer’s Disease Accompanied by Decreased PSD-95 Fluorescence. Am. J. Pathol..

[7] Shi Y., Wu Q., Wang X. (2021). Modeling brain development and diseases with human cerebral organoids. Curr. Opin. Neurobiol..

[8] Mayer M.G., Fischer T. (2024). Microglia at the blood brain barrier in health and disease. Front. Cell. Neurosci..

[9] Eskola M., Kos G., Elliott C.T., Hajšlová J., Mayar S., Krska R. (2020). Worldwide contamination of food-crops with mycotoxins: Validity of the widely cited ‘FAO estimate’ of 25%. Crit. Rev. Food Sci. Nutr..

[10] Gupta R.C., Srivastava A., Lall R. (2018). Ochratoxins and Citrinin. Veterinary Toxicology: Basic and Clinical Principles.

[11] Ladeira C., Viegas C., Pinheiro A.C., Sabino R., Viegas S., Brandão J., Veríssimo C. (2016). Mycotoxins: Genotoxicity Studies and Methodologies. Environmental Mycology in Public Health: Fungi and Mycotoxins Risk Assessment and Management.

[12] Vettorazzi A., Gonzalez-Penas E., Troconiz I.F., Arbillaga L., Corcuera L.A., Gil A.G., de Cerain A.L. (2009). A different kinetic profile of ochratoxin A in mature male rats. Food Chem. Toxicol..

[13] Han Z., Zhao Z., Shi J., Liao Y., Zhao Z., Zhang D., Wu Y., De Saeger S., Wu A. (2013). Combinatorial approach of LC-MS/MS and LC-TOF-MS for uncovering in vivo kinetics and biotransformation of ochratoxin A in rat. J. Chromatogr. B Anal. Technol. Biomed. Life Sci..

[14] Kaya E.M.Ö., Korkmaz O.T., Uğur D.Y., Şener E., Tunçel A.N., Tunçel M. (2019). Determination of Ochratoxin-A in the brain microdialysates and plasma of awake, freely moving rats using ultra high performance liquid chromatography fluorescence detection method. J. Chromatogr. B Anal. Technol. Biomed. Life Sci..

[15] Wu T.Y., Khorramshahi T., Taylor L.A., Bansal N.S., Rodriguez B., Rey I.R. (2022). Prevalence of Aspergillus-Derived Mycotoxins (Ochratoxin, Aflatoxin, and Gliotoxin) and Their Distribution in the Urinalysis of ME/CFS Patients. Int. J. Environ. Res. Public Health.

[16] Alonso-Garrido M., Frangiamone M., Font G., Cimbalo A., Manyes L. (2021). In vitro blood brain barrier exposure to mycotoxins and carotenoids pumpkin extract alters mitochondrial gene expression and oxidative stress. Food Chem. Toxicol..

[17] Babayan N., Tadevosyan G., Khondkaryan L., Grigoryan R., Sarkisyan N., Haroutiounian R., Stopper H. (2020). Ochratoxin A induces global DNA hypomethylation and oxidative stress in neuronal cells in vitro. Mycotoxin Res..

[18] Behrens M., Hüwel S., Galla H.J., Humpf H.U. (2015). Blood-brain barrier effects of the fusarium mycotoxins deoxynivalenol, 3 acetyldeoxynivalenol, and moniliformin and their transfer to the brain. PLoS ONE.

[19] Bhat P.V., Pandareesh M.D., Khanum F., Tamatam A. (2016). Cytotoxic Effects of Ochratoxin A in Neuro-2a Cells: Role of Oxidative Stress Evidenced by N-acetylcysteine. Front. Microbiol..

[20] Chansawhang A., Phochantachinda S., Temviriyanukul P., Chantong B. (2022). Corticosterone potentiates ochratoxin A-induced microglial activation. Biomol. Concepts.

[21] Chu C.S., Chen Y.T., Liang W.Z. (2024). Investigation of the mechanisms behind ochratoxin A-induced cytotoxicity in human astrocytes and the protective effects of N-acetylcysteine. J. Appl. Toxicol..

[22] Fu M., Chen Y., Yang A. (2024). Ochratoxin A induces mitochondrial dysfunction, oxidative stress, and apoptosis of retinal ganglion cells (RGCs), leading to retinal damage in mice. Int. Ophthalmol..

[23] Park S., Lim W., You S., Song G. (2019). Ochratoxin A exerts neurotoxicity in human astrocytes through mitochondria-dependent apoptosis and intracellular calcium overload. Toxicol. Lett..

[24] Penalva-Olcina R., Juan C., Fernández-Franzón M., Juan-García A. (2024). Involvement of pro-inflammatory mediators and cell cycle disruption in neuronal cells induced by gliotoxin and ochratoxin A after individual and combined exposure. Toxicol. Lett..

[25] Penalva-Olcina R., Juan C., Fernández-Franzón M., Juan-García A. (2023). Cell cycle and enzymatic activity alterations induced by ROS production in human neuroblastoma cells SH-SY5Y exposed to fumonisin B1, ochratoxin A and their combination. Toxicol. Vitr..

[26] Radzka-Pogoda A., Radzki R.P., Bieńko M., Szponar J., Sokołowska B., Kulik A., Lewicka M., BorzęCki A. (2023). Ochratoxin A and Aflatoxin B1 as Factors of Bone Damage and Neurodegeneration Through the Influence on the Immunomodulation Processes of TNF-α and IL-6 Concentrations. Pol. Hyperb. Res..

[27] Sharma R., Gettings S.M., Hazell G., Bourbia N. (2023). In vitro study of ochratoxin A in the expression of genes associated with neuron survival and viability. Toxicology.

[28] Tsilioni I., Theoharides T.C. (2024). Ochratoxin A stimulates release of IL-1β, IL–18 and CXCL8 from cultured human microglia. Toxicology.

[29] Zingales V., Piunti C., Micheli S., Cimetta E., Ruiz M.J. (2024). Development of an Easy-To-Use Microfluidic System to Assess Dynamic Exposure to Mycotoxins in 3D Culture Models: Evaluation of Ochratoxin A and Patulin Cytotoxicity. Foods.

[30] Brahmi M., Adli D.E.H., Kaoudj I., Alkholifi F.K., Arabi W., Sohbi S., Ziani K., Kahloula K., Slimani M., Sweilam S.H. (2025). Chemical Composition, In Vivo, and In Silico Molecular Docking Studies of the Effect of *Syzygium aromaticum* (Clove) Essential Oil on Ochratoxin A-Induced Acute Neurotoxicity. Plants.

[31] Izco M., Vettorazzi A., Forcen R., Blesa J., de Toro M., Alvarez-Herrera N., Cooper J.M., Gonzalez-Peñas E., de Cerain A.L., Alvarez-Erviti L. (2021). Oral subchronic exposure to the mycotoxin ochratoxin A induces key pathological features of Parkinson’s disease in mice six months after the end of the treatment. Food Chem. Toxicol. Int. J. Publ. Br. Ind. Biol. Res. Assoc..

[32] Mateo E., Tonino R.P.B., Canto A., Noyola A.M., Miranda M., Soria J.M., Esparza M.A.G. (2022). The Neurotoxic Effect of Ochratoxin-A on the Hippocampal Neurogenic Niche of Adult Mouse Brain. Toxins.

[33] Paradells S., Rocamonde B., Llinares C., Herranz-Pérez V., Jimenez M., Garcia-Verdugo J.M., Zipancic I., Soria J.M., Garcia-Esparza M.A. (2015). Neurotoxic effects of ochratoxin A on the subventricular zone of adult mouse brain. J. Appl. Toxicol. JAT.

[34] Tschirren L., Siebenmann S., Pietsch C. (2018). Toxicity of ochratoxin to early life stages of zebrafish (*Danio rerio*). Toxins.

[35] Valadas J., Sachett A., Marcon M., Bastos L.M., Piato A. (2021). Ochratoxin A induces behavioral and neurochemical changes in adult zebrafish. BioRxiv Prepr. Serv. Biol..

[36] Wu T.S., Lin Y.T., Huang Y.T., Yu F.Y., Liu B.H. (2020). Ochratoxin A triggered intracerebral hemorrhage in embryonic zebrafish: Involvement of microRNA-731 and prolactin receptor. Chemosphere.

[37] Bhat P.V., Anand T., Manu T.M., Khanum F. (2018). Restorative effect of L-Dopa treatment against Ochratoxin A induced neurotoxicity. Neurochem. Int..

[38] Serrano-Civantos M., Beraza E., Álvarez-Erviti L., de Cerain A.L., Vettorazzi A. (2025). Potential role of ochratoxin A in Parkinson’s disease: A systematic review of current evidence. Arch. Toxicol..

[39] Bhatt S., Puli L., Patil C.R. (2021). Role of reactive oxygen species in the progression of Alzheimer’s disease. Drug Discov. Today.

[40] Penalva-Olcina R., Juan C., Fernández-Franzón M., Juan-García A. (2025). Neurotoxic implications of gliotoxin and ochratoxin A in SH-SY5Y cells: ROS-induced apoptosis and genotoxicity. Toxicol. Lett..

[41] Cheignon C., Tomas M., Bonnefont-Rousselot D., Faller P., Hureau C., Collin F. (2018). Oxidative stress and the amyloid beta peptide in Alzheimer’s disease. Redox Biol..

[42] Gorantla N.V., Chinnathambi S. (2021). Autophagic Pathways to Clear the Tau Aggregates in Alzheimer’s Disease. Cell. Mol. Neurobiol..

[43] Silva N.M.L.E., Gonçalves R.A., Pascoal T.A., Lima-Filho R.A.S., Resende E.D.P.F., Vieira E.L.M., Teixeira A.L., de Souza L.C., Peny J.A., Fortuna J.T.S. (2021). Pro-inflammatory interleukin-6 signaling links cognitive impairments and peripheral metabolic alterations in Alzheimer’s disease. Transl. Psychiatry.

[44] Dong Y., Yu H., Li X., Bian K., Zheng Y., Dai M., Feng X., Sun Y., He Y., Yu B. (2022). Hyperphosphorylated tau mediates neuronal death by inducing necroptosis and inflammation in Alzheimer’s disease. J. Neuroinflammation.

[45] Brown J., Reading S.J., Jones S., Fitchett C.J., Howl J., Martin A., Longland C.L., Michelangeli F., Dubrova Y.E., Brown C.A. (2000). Critical evaluation of ECV304 as a human endothelial cell model defined by genetic analysis and functional responses: A comparison with the human bladder cancer derived epithelial cell line T24/83. Lab. Investig. A J. Tech. Methods Pathol..

[46] Wang D., Chen F., Han Z., Yin Z., Ge X., Lei P. (2021). Relationship Between Amyloid-β Deposition and Blood–Brain Barrier Dysfunction in Alzheimer’s Disease. Front. Cell. Neurosci..

[47] Fakorede S., Lateef O.M., Garuba W.A., Akosile P.O., A Okon D., Aborode A.T. (2025). Dual impact of neuroinflammation on cognitive and motor impairments in Alzheimer’s disease. J. Alzheimer’s Dis. Rep..

[48] Singh A., Kukreti R., Saso L., Kukreti S. (2019). Oxidative Stress: A Key Modulator in Neurodegenerative Diseases. Molecules.

[49] Gylys K.H., A Fein J., Wiley D.J., Cole G.M. (2004). Rapid annexin-V labeling in synaptosomes. Neurochem. Int..

[50] Lleó A., Núñez-Llaves R., Alcolea D., Chiva C., Balateu-Paños D., Colom-Cadena M., Gomez-Giro G., Muñoz L., Querol-Vilaseca M., Pegueroles J. (2019). Changes in Synaptic Proteins Precede Neurodegeneration Markers in Preclinical Alzheimer’s Disease Cerebrospinal Fluid. Mol. Cell. Proteom. MCP.

[51] Hampel H., Mesulam M.-M., Cuello A.C., Khachaturian A.S., Vergallo A., Farlow M.R., Snyder P.J., Giacobini E., Khachaturian Z.S. (2019). Revisiting the Cholinergic Hypothesis in Alzheimer’s Disease: Emerging Evidence from Translational and Clinical Research. J. Prev. Alzheimer’s Dis..

[52] Arce-López B., Lizarraga E., Vettorazzi A., González-Peñas E. (2020). Human Biomonitoring of Mycotoxins in Blood, Plasma and Serum in Recent Years: A Review. Toxins.

[53] Kőszegi T., Poór M. (2016). Ochratoxin A: Molecular Interactions, Mechanisms of Toxicity and Prevention at the Molecular Level. Toxins.

